# Poor sleep quality and associated factors among adult chronic kidney disease patients

**DOI:** 10.3389/fmed.2024.1366010

**Published:** 2024-05-01

**Authors:** Yibeltal Yismaw Gela, Liknaw Workie Limenh, Wudneh Simegn, Wondim Ayenew, Gashaw Sisay Chanie, Abdulwase Mohammed Seid, Alemante Tafese Beyna, Dereje Esubalew, Melese Legesse Mitku, Assefa Kebad Mengesha, Mihret Melese

**Affiliations:** ^1^Department of Physiology, College of Medicine and Health Sciences, University of Gondar, Gondar, Ethiopia; ^2^Department of Pharmaceutics, School of Pharmacy, College of Medicine and Health Sciences, University of Gondar, Gondar, Ethiopia; ^3^Department of Social and Administrative Pharmacy, School of Pharmacy, College of Medicine and Health Sciences, University of Gondar, Gondar, Ethiopia; ^4^Department of Clinical Pharmacy, School of Pharmacy, College of Medicine and Health Sciences, University of Gondar, Gondar, Ethiopia; ^5^Department of Pharmacology, School of Pharmacy, College of Medicine and Health Sciences, University of Gondar, Gondar, Ethiopia; ^6^Department of Human Physiology, College of Medicine and Health Sciences, Ambo University, Ambo, Ethiopia; ^7^Department of Pharmaceutical Chemistry, School of Pharmacy, College of Medicine and Health Sciences, University of Gondar, Gondar, Ethiopia

**Keywords:** poor sleep quality, chronic kidney disease, PSQI, Ethiopia, cross-sectional study

## Abstract

**Background:**

Poor sleep quality is a common concern in chronic kidney disease (CKD) patients, which can accelerate the progression of chronic renal disease and negatively impact their health-related quality of life, potentially leading to greater morbidity and mortality rates. It can also have an effect on the immune system, cognitive function, and emotional well-being of CKD patients. Furthermore, poor sleep quality may contribute to drug noncompliance and decreased participation in the entire treatment plan. Nonetheless, no research has been undertaken in Ethiopia on the prevalence of poor sleep quality and its associated factors among CKD patients.

**Objective:**

This study aimed to assess the prevalence of poor quality of sleep and associated factors among chronic kidney disease patients at the University of Gondar Comprehensive Specialized and Felege Hiwot Referral Hospitals in 2020.

**Methods:**

A cross-sectional study design was implemented at the University of Gondar Comprehensive Specialized and Felege Hiwot Referral Hospitals between February and April 2020. The study participants were chosen through systematic random sampling techniques. The Pittsburgh Sleep Quality Index (PSQI), a validated assessment tool, was utilized to measure sleep quality. A PSQI total score > 5 was used as an indicator of poor sleep quality. Subsequently, the data obtained were entered into Epi Data version 3.0 and then transferred to STATA 14 for analysis. Both bivariable and multivariable binary logistic regression analyses were performed to recognize factors associated with poor sleep quality. In the multivariable logistic regression analysis, variables demonstrating a *p*-value of ≤0.05 were considered statistically associated to poor sleep quality.

**Results:**

In this study, 424 CKD patients were included. Among screened CKD patients, 42.9% tested positive for poor sleep quality with a 95% CI (38 to 47%). Independent predictors of poor sleep quality among CKD patients were common mental disorder [AOR = 1.8, 95% CI (1.19–2.89)], anemia [AOR = 2.7, 95% CI (1.71–4.36)], declined eGFR between 60 and 89.9 [AOR = 1.6; 95% CI (2.28–5.54)], 30–59.9 [AOR = 2.6, 95% CI (1.53–4.43)], and ≤ 30 [AOR = 3.8, 95% CI (1.17–12.61)], age > 50 years [AOR = 1.7(1.11–2.69)] and duration of disease 2.9 [AOR = 2.9, 95% CI (1.77–4.90)].

**Conclusion:**

In our study, almost 1 out of 2 CKD patients assessed for poor sleep quality tested positive. It was noted that poor sleep quality was more frequent among CKD patients with common mental disorders, anemia, decreased eGFR levels, individuals aged over 50 years, and those with a longer duration of the disease. Consequently, it’s advised to regularly screen these CKD patients for poor sleep quality.

## Introduction

Chronic kidney disease (CKD) is defined by chronic and permanent kidney deterioration, resulting in the kidney’s incapacity to function (1). The prevalence of CKD has recently been quickly growing as a result of higher rates of hypertension, diabetes, and obesity (2). According to institutional research study done in Ethiopia, the prevalence of CKD ranges from 22.1 to 38.6% (3–5).

Poor quality of sleep is difficulty initiating or maintaining sleep, waking up too early or unrefreshing sleep, despite adequate opportunity and circumstances for sleep, leading to impairment of daytime function (6). Sleep disorders present frequently with CKD with substantial impact on both the patient and the health system (7).

The prevalence of poor sleep quality in patients with CKD ranges from 37 to 97%, with the highest prevalence observed among hemodialysis patients in Turkey (8–13). Low-quality sleep can hasten the progression of CKD (14, 15) as well as have a negative influence on health-related quality of life (16), resulting in increased morbidity and death (17–20). It also has an impact on CKD patients’ immune systems, cognitive capabilities, and emotional well-being (21). Furthermore, poor sleep quality might lead to drug noncompliance and decreased engagement in the overall treatment plan (22, 23).

Healthcare providers often overlook sleep disorders (24), which are common but frequently unnoticed in chronic kidney disease patients (6). There have been no studies conducted in Ethiopia to determine the prevalence of poor sleep quality and its contributing factors among CKD patients. Therefore, this study aims to assess the prevalence of poor sleep quality and associated factors among CKD patients.

## Methods and materials

### Study setting and period

An institution-based cross-sectional study was conducted from February to April, 2020 at the University of Gondar Comprehensive Specialized Hospital and Felege Hiwot Referral Hospital.

### Study population

During the data collecting period, all adult CKD patients encountered at the University of Gondar Comprehensive Specialized and Felege Hiwot Referral Hospitals were included in the study. However, CKD patients on dialysis were excluded from the study.

### Sample size calculation and sampling procedure

The sample size was calculated using the following single proportion formula, N= $\left[\frac{\;{\left(Z_{\alpha /2}\right)}^{2}\times p\left(1-p\right)\;}{d^{2}}\right]$ = $\left[\frac{{\left(1.96\right)}^{2}\times 0.5\left(1-0.5\right)\;}{{\left(0.05\right)}^{2}}\right]$ = 385, where N: sample size, p: estimated prevalence value (50%), d: margin of sampling error tolerated (5%), Z_α/2_ (1.96): critical value at 95% confidence interval of certainty.

After adding 10% of the non-response rate, a total of 424 chronic kidney disease patients were selected.

**Figure 1:**
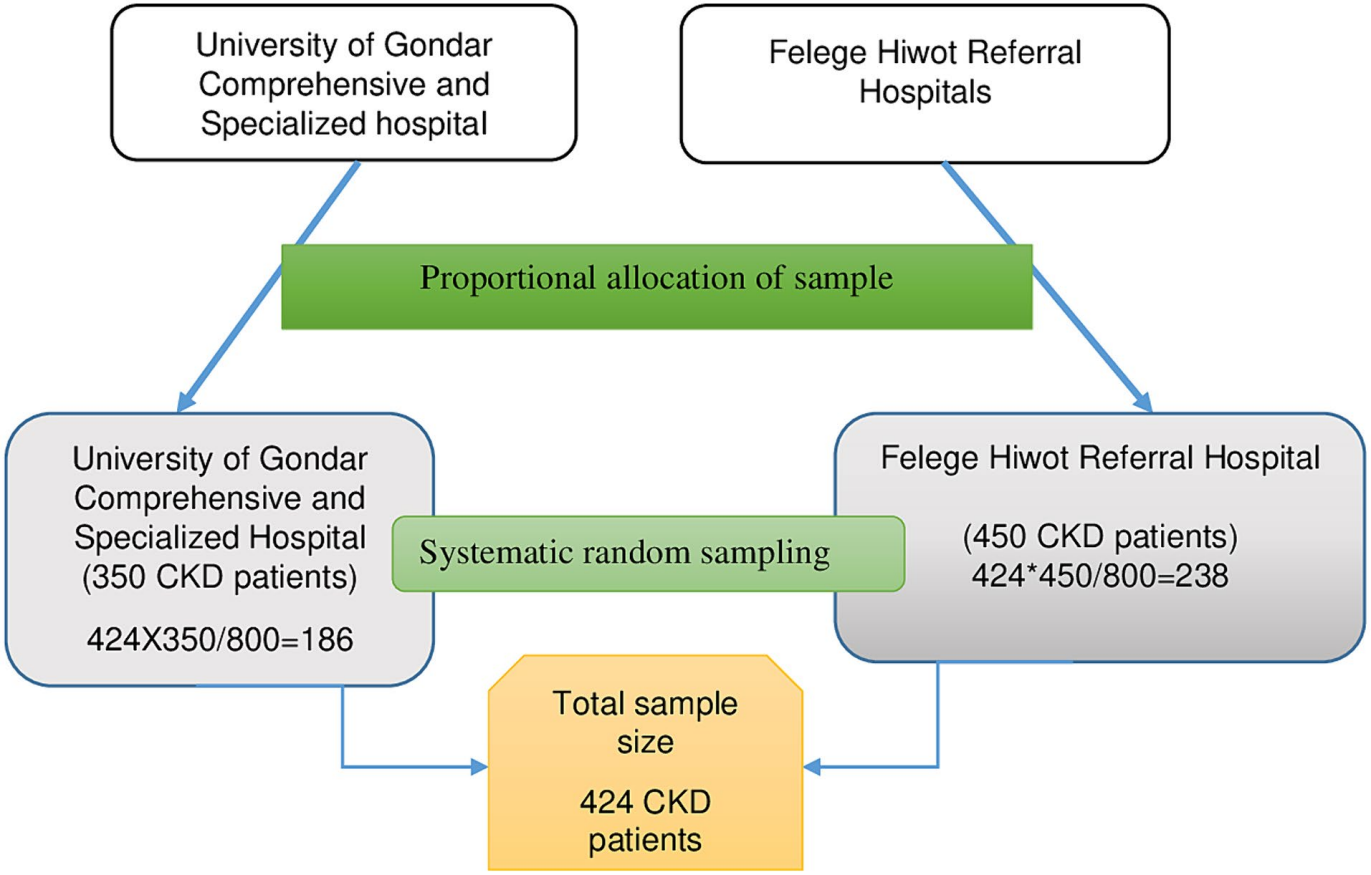
Illustrates the sampling process used to select chronic kidney disease patients at the University of Gondar Comprehensive Specialized Hospital and Felege Hiwot Referral Hospital, 2020.

During the data collection period, 350 patients were seen at the follow-up clinic of the University of Gondar Comprehensive Specialized Hospital, while 450 patients were encountered at the Felege Hiwot Referral Hospital. Employing the proportionate random sampling technique, 186 CKD patients were chosen from the University of Gondar Comprehensive Specialized Hospital, and 238 from the Felege Hiwot Referral Hospital. A total of 424 CKD patients were recruited using systematic random sampling, employing a K value of 2. The initial participant was selected using a lottery method, followed by every second patient for the interviews (Figure 1).

### Operational definitions

Chronic kidney disease (CKD) is characterized by a sustained abnormality in kidney structure or function lasting for at least 3 months. This may manifest as a glomerular filtration rate (GFR) below 60 mL/min/1.73 m^2^, albuminuria (urine albumin ≥30 mg per 24 h or urine albumin-to-creatinine ratio [ACR] ≥30 mg/g), abnormalities in urine sediment, histology, or imaging indicating kidney damage, renal tubular disorders, or a history of kidney transplantation (1, 25).

The estimated glomerular filtration rate (eGFR) was calculated using epidemiology of collaboration (EPI) equations (26, 27).

Quality of sleep was screened using Pittsburgh Sleep Quality Index with optimal cut-off scores above 5 (28).

Physical activity: according to World Health Organization (WHO) is adults and older engage in 150–300 min of moderate-intensity aerobic physical activity per week, or 75–150 min of vigorous-intensity aerobic physical activity throughout the week (29).

Common mental disorders (CMDs) was screened using SRQ-F (Self-Reporting Questionnaire-Falk Institute) which contains 29 items. Those patients who scored ≥8 out of 29 scores in the last 1 month were screened as positive for CMD (30).

Social support is assistance provided when financial, social, or psychological problems arise. The Oslo Social Support Scale tool was used to screen social support status, which includes three items scored out of 14 and is defined as weak support (3–8), moderate support (9–11), and strong support (12–14) depending on the results (31).

Anemia is defined as having a hemoglobin concentration below 13 g/dL in men and below 12 g/dL in women (32).

Comorbidity: the presence of chronic kidney disease and one or more of the following diseases: HIV/AIDS, hypertension, cardiovascular diseases, and diabetic mellitus.

Participants were categorized based on their body mass index (BMI) into different groups: those with a BMI of ≤18.5 kg/m^2^ were classified as underweight, individuals with a BMI between 18.5 kg/m^2^ and 24.9 kg/m^2^ were categorized as having a normal weight, and those with a BMI between 25 kg/m^2^ and 29.9 kg/m^2^ were classified as overweight. A BMI exceeding 30 kg/m^2^ was considered indicative of obesity (33, 34).

### Data collection procedure and tools

Data were collected using interviewer-administered structured questionnaire which consists of sociodemographic characteristics, Medical Record Review, physical measurements (weight and height), Pittsburgh Sleep Quality Index and Oslo Social Support Scale.

The Pittsburgh Sleep Quality Index (PSQI) is a validated assessment tool used to evaluate sleep quality in Ethiopia (30). It consists of 19 items organized into seven components, assessing sleep duration, efficiency, latency, disturbances, daytime dysfunction, frequency of sleep medication use, and subjective sleep quality. Each item is rated on a scale of 0 to 3. The scores from these components are combined to generate a total score ranging from 0 to 21. A higher total score, known as the global score, indicates lower sleep quality. A PSQI global score of >5 is used as a cutoff to identify poor sleep quality, with a sensitivity of 89.6% and specificity of 86.5% (35).

### Data analysis procedure

The data undertook cleaning, coding, and entry into Epi-Data 3. Subsequently, it was exported to STATA 14 for analysis. Continuous variables were represented using the mean and standard deviation, while categorical variables were described through frequency distribution and pie charts.

Both bi-variable and multi-variable logistic regression analyses were conducted. In the bi-variable logistic regression, variables displaying an association with poor sleep quality at *p* ≤ 0.25 were included in the multi-variable regression model. Within the multi-variable logistic regression, variables demonstrating a *p*-value of ≤0.05 with a 95% confidence interval were considered significantly associated with poor sleep quality. The model’s fitness was assessed using the Hosmer and Lemeshow test; the model was considered fitted as the value obtained (0.12) exceeded the threshold of 0.05.

Furthermore, to ascertain the reliability of the Pittsburgh Sleep Quality Index, the Cronbach’s alpha test was conducted, yielding a reliability coefficient of 0.79. This value indicates good reliability of the tool in measuring sleep quality.

### Data quality management

The questionnaire was translated into the Amharic language by a language expert, followed by retranslation into English by another expert to ensure consistency. The principal investigator provided training to the data collectors regarding the Pittsburgh Sleep Quality Index, Oslo Social Support Scale, Medical Record Review, as well as the procedure for measuring height and weight.

## Results

### Sociodemographic characteristic of study participants

This research involved 424 patients diagnosed with chronic kidney disease. The average age of the patients was 53.8 years, with a standard deviation of 16.8 years. Among the CKD patients, the majority, comprising 256 individuals (60.4%), were male. Additionally, 177 participants (41.6%) had attended primary school, 222 (52.5%) were employed, 321 (75.7%) practiced Orthodox Christianity, and 333 (78.5%), were married (Table 1).

**Table 1:** Socio-demographic characteristics of chronic kidney disease patients at the University of Gondar Comprehensive Specialized and Felege Hiwot Referral Hospitals, 2020.

Variables	Categories	Number (%)
Age (year)	<50	154 (36.3)
	≥50	270 (63.7)
Sex	Male	256 (60.4)
	Female	168 (39.6)
Religion	Orthodox	321 (75.7)
	Muslim	72 (17)
	Protestant	31 (7.3)
Occupation	Employed	222(52.5)
	Merchant	71(16.7)
	Farmer	71(17.5)
	Housewife	57(13.2)
Educational level	Unable to read and write	88 (20.7)
	≤8 grade	177 (41.6)
	Grade 9–12	72 (17)
	College and above	87 (20.5)
Marital status	Single	54 (12.7)
	Married	333 (78.5)
	Divorced	18 (4.3)
	Widowed	19 (4.5)
Income (ETB)	≤1,500	130 (30.7)
	1,501–3500	107 (25.2)
	≥3,501	187 (44.1)
Residence	Urban	335 (79.0)
	Rural	89 (21)
BMI (Kg/m^2^)	Normal	345 (81.4)
	Underweight	42 (9.9)
	Overweight	37(8.7)

BMI = Body Mass Index; ETB = Ethiopia Birr.

### Clinical and psychosocial related factors of CKD patients

The mean creatinine level among CKD patients were 1.8 mg/dL (SD ± 1.4). One hundred nighty three (45%) patients had eGFR ≥90 mL/min/m^2^ and 119 (28.1%) of them were anemic. At the time of data collection, 54% of CKD patients’ duration since diagnosis was 5 years and less. Two hundred ninety-one (68.6%) CKD patients had comorbidities (Table 2).

**Table 2:** Clinical characteristics of chronic kidney disease patients at the University of Gondar Comprehensive Specialized and Felege Hiwot Referral Hospitals, 2020.

Variables	Categories	Number (%)
Creatinine(mg/dl)(Mean ± SD)	1.8	1.8 ± 1.4
eGFR (ml/min/m^2^)	≥90	192 (45.3)
	60–89.9	117(27.6)
	30–59.9	103 (24.3)
	≤30	12(2.8)
Anemia	No	305 (71.9)
	Yes	119 (28.1)
Social support	Poor social support	189 (44.6)
	Moderate support	144 (34)
	Strong support	91 (21.5)
Duration of CKD (year)	≤5 year	229 (54)
	>5 year	195 (46)
Comorbidity	No	133 (31.4)
	Yes	291 (68.6)

eGFR = estimated glomerular filtration rate; CKD Chronic Kidney Disease.

### Prevalence of poor sleep quality among CKD patients

**Figure 2:**
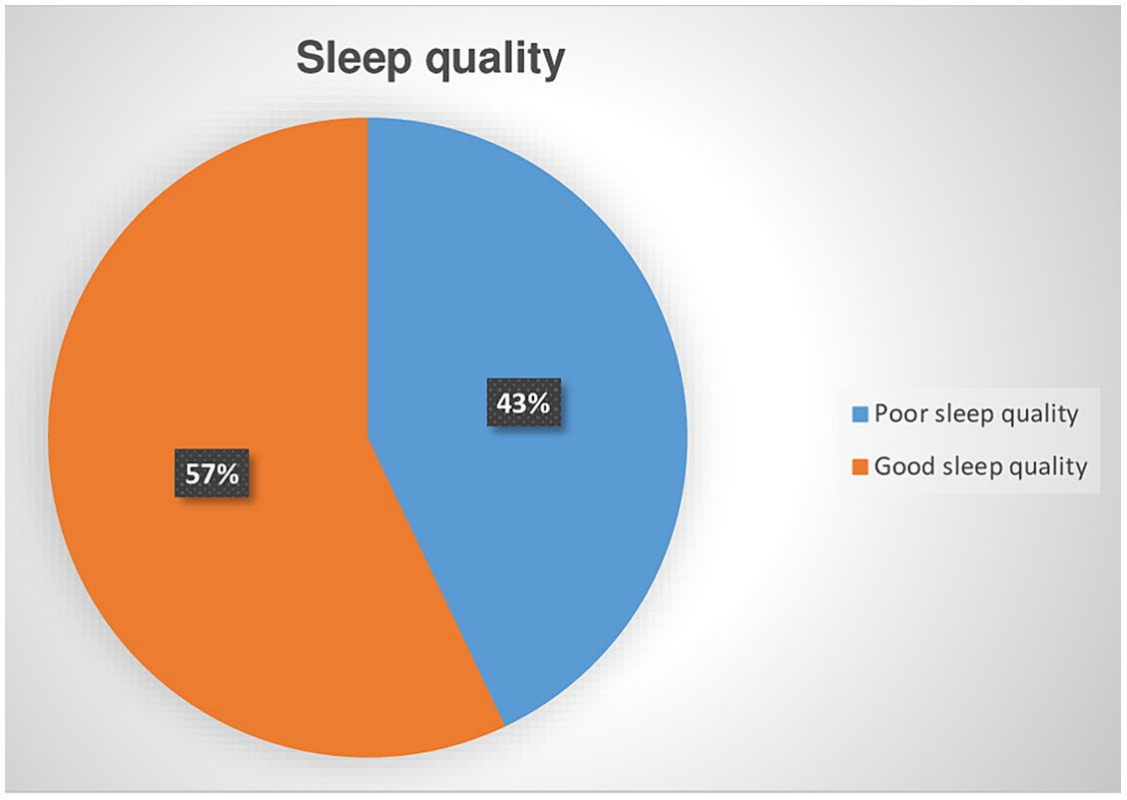
Prevalence of poor sleep quality among chronic kidney disease patients at the University of Gondar Comprehensive Specialized and Felege Hiwot Referral Hospitals in 2020.

Among screened CKD patients for poor sleep quality, 182 (42.9%) was positive for poor quality of sleep with a 95% CI (38 to 47%). (Figure 2). The value of Pittsburgh Sleep Quality Index (PSQI), Mean ± SD was 4.8 ± 6.11.

### Predictors of poor sleep quality among CKD patients

Among variables entered into bivariable logistic regression, common mental disorder, duration of CKD, anemia, physical activity, estimated glomerular filtration rate (eGFR), and age were associated with poor quality of sleep at *p*-value of ≤0.25. However, in multi-variable logistic regression analysis anemia, common mental disorders, disease duration, eGFR, and age were variables significantly associated with poor quality of sleep at p-value of ≤0.05.

The odds of poor sleep quality among CKD patients with common mental disorders were 1.8 times higher compared to CKD patients without common mental disorders (AOR = 1.8, 95% CI 1.19–2.89). Among anemic CKD patients, the odds of poor sleep quality were 2.7 times higher compared to non-anemic CKD patients (AOR = 2.7, 95% CI 1.71–4.36).

The odds of poor sleep quality among CKD patients with eGFR between 60 and 89.9 mL/min/m2, 30–59.9 mL/min/m2, and ≤ 30 mL/min/m2 were 2.9 (AOR = 2.9, 95% CI 1.77–4.90), 2.6 (AOR = 2.6, 95% CI 1.53–4.43), and 3.8 (AOR = 3.8, 95% CI 1.17–12.61) times higher, respectively, compared to CKD patients with eGFR ≥90 mL/min/m2.

Among CKD patients, the odds of poor sleep quality were 1.7 times higher for those above 50 years old compared to those ≤50 years old (AOR = 1.7, 95% CI 1.11–2.69). Additionally, the odds of poor sleep quality were 1.6 times higher for CKD patients with a duration of over 5 years compared to those with a duration ≤5 years (AOR = 1.6, 95% CI 1.03–2.46) (Table 3).

**Table 3:** Factors associated with poor quality of sleep among CKD patients at the University of Gondar Comprehensive Specialized and Felege Hiwot Referral Hospitals, 2020.

		Poor sleep quality			OR(95% CI)	
Variables	Categories	Total (*N*) %	Yes (*N*) %	No (*N*) %	COR	AOR
Common mental disorder	No	259(61.1)	94(22.1)	165(38.9)	1	1
	Yes	165(38.9)	88(20.7)	77(18.2)	2.0(1.35–2.98)	1.8(1.19–2.89)^*^
Anemia	No	305 (71.9)	109 (46.2)	196 (25.7)	1	1
	Yes	119 (28.1)	46(10.8)	73(17.2)	2.8(1.84–4.41)	2.7(1.71–4.36)^*^
eGFR (ml/min/m^2^)	> = 90	192 (45.3)	56 (13.2)	136(32.1)	1	1
	60–89.9	117(27.6)	67(15.8)	50(11.8)	3.2(2.01–5.26)	2.9(1.77–4.90)^*^
	30–59.9	103 (24.3)	52(12.3)	51(12.0)	2.5(1.50–4.06)	2.6(1.53–4.43)^*^
	≤30	12(2.8)	7(1.6)	5(1.2)	3.4(1.03–11.16)	3.8(1.17–12.61)^*^
Duration of disease (year)	≤5	229 (54)	90(21.2)	139(32.8)	1	1
	>5	195(46)	103 (24.3)	84(21.7)	1.4(0.93–2.03)	1.6(1.03–2.46)^*^
Physical activity (Min/week)	≥150	84(19.8)	31(7.3)	53(12.5)	1	1
	<150	340 (80.2)	151 (35.6)	189 (44.6)	1.4(0.83–2.23)	1.3(0.76–2.22)
Age	<50	154(36.3)	55(13)	99 (23.3)	1	1
	≥50	270 (63.7)	127 (30)	143(33.7)	1.6(1.06–2.40)	1.7(1.11–2.69)^*^

eGFR = estimated glomerular filtration rate; CKD = Chronic Kidney Disease; CI = Confidence Interval, COR = Crude Odds Ratio, eGFR = Estimated glomerular filtration rate, OR = Odds Ratio, N = Number, *p value ≤ 0.05.

## Discussion

The purpose of this study was to determine the prevalence of poor sleep quality and contributing factors among CKD patients in Ethiopia. Nearly half of the CKD patients had poor sleep quality. Age, anemia, disease duration, and common mental disorder all had significant associations with poor sleep quality in CKD patients.

In our study, the prevalence of poor quality of sleep was found to be 42.9% with 95% CI (38 to 47%), which was similar to the reported prevalence from Turkey (42.2%) (18), but higher than the prevalence observed in two other studies from Saudi Arabia (36.4%) (22), and Japan (37%) (36). Possible reasons for these variations could be attributed to differences in sample sizes, with Saudi Arabia having a smaller sample size, and variations in the assessment tool’s cutoff value, such as Japan using a cutoff of ≥6 to evaluate sleep quality.

The observed prevalence in this study was less compared to Nigeria (50.2%) (8) and China (66.4%) (37), both of which involved solely stage 3–5 CKD patients, as well as Pakistan (65.8%) (38) and India (86.5%) (39). In the Pakistan and India studies, patients undergoing dialysis were also included, and they utilized a PSQI cutoff value of ≥5 to define poor sleep quality, potentially contributing to the higher prevalence of poor sleep quality in those studies.

A lower estimated glomerular filtration rate (eGFR) has been established as a predictor of poor sleep quality in people with CKD, correlating with prior research findings (8, 37). The factors that contribute to sleep problems in CKD patients are numerous and complex. The impairment of baroreceptor reflex function is a common problem in CKD. This defect causes an imbalance in sympathetic and vagal activity, resulting in increased sympathetic nervous system activity and decreased vagal tone (40). Normally, there is a natural decrease in sympathetic activity and a rise in vagal tone during sleep, which is interrupted in CKD patients (41). Chronic inflammation and stimulation of the hypothalamic–pituitary–adrenal axis and sympathetic nervous system are frequent in CKD patients (42). These factors have been linked to sleep disruptions. Furthermore, uremic toxin buildup in the cerebrovascular circulation in CKD may disturb the normal functioning of brain cells involved in sleep induction (43). The hypothalamus and basal forebrain play a crucial role in regulating sleep–wake cycles and are sensitive to changes in metabolic and hormonal status, often disrupted in chronic kidney disease (CKD). The activity of the c-fos protein (FOS) in ventrolateral preoptic neurons (VLPO) of the hypothalamus is associated with the sleep pattern. The expression of FOS in VLPO neurons of the hypothalamus is linked to the activation of neurons that initiate sleep, and this expression is affected by uremia in CKD patients, resulting in sleep disturbances. FOS protein, an immediate-early gene product, is found in a group of ventrolateral preoptic neurons specifically activated during sleep (44, 45).

Patients with end-stage kidney disease (ESKD) have higher amounts of orexin, a neuropeptide that increases alertness, which may contribute to poor sleep quality (46). Furthermore, CKD patients have lower amounts of melatonin, a hormone important in regulating sleep–wake cycles (41, 47).

In summary, the numerous causes of poor sleep quality in CKD patients include uremic toxins, disruption in circadian rhythms, fluid imbalance, electrolyte imbalance, changes in hormone balance, activation of sympathetic nervous system and inflammation. As the disease duration increases patients with CKD are more likely to develop poor sleep quality.

The presence of anemia in CKD patients was significantly associated with a poor quality of sleep similar with others studies (38, 48). The exact underlying mechanisms for the association of anemia and poor sleep quality remain unclear. One potential explanation is that anemia could increase insomnia risk through a shared gene (48), fatigue as a symptom of anemia may induce sleep problems (49). Another potential mechanism of this phenomenon anemia causes a decrease in the blood’s oxygen-carrying capacity (50). Hypoxia can alter the brain’s regulatory centers involved in sleep–wake cycles, thereby causing sleep disorders (50). Again anemic people may suffer increased respiratory effort during sleep, a condition known as sleep-disordered breathing. This greater effort may be caused by the body’s attempt to compensate for low oxygen (51).

Another factor associated with poor sleep quality was older age, which was supported by other studies (9, 18, 22, 37, 52). Aging is associated with decreased ability to maintain sleep, reduced nocturnal sleep duration, and decreased deep sleep (53).

Older adults often experience an increased frequency of awakenings during the night. These awakenings may occur due to a variety of reasons, including changes in bladder function leading to more frequent trips to the bathroom, discomfort from various health conditions, or a decrease in the ability to maintain deep sleep (9). Older individuals may find it challenging to return to sleep after waking during the night. Factors such as pain, discomfort, medication side effects, or underlying health conditions can contribute to prolonged periods of wakefulness during the night. While the need for sleep remains relatively constant throughout adulthood, older adults often experience a reduction in the total duration of sleep at night. This reduction can be influenced by changes in sleep architecture, more frequent awakenings, or a shift in the circadian rhythm (53).

Common mental disorder was significantly associated with poor quality of sleep which is supported by other studies (37, 38). Common mental disorders, which often coincide with poor sleep quality, frequently exhibit changes in neurotransmitter levels specifically serotonin, dopamine, and norepinephrine as well as disruptions in the hypothalamic–pituitary–adrenal (HPA) axis and heightened reactivity to stressors. These factors contribute to the sleep disturbances commonly observed in individuals with these mental health conditions.

In individuals experiencing depression, disruptions in the circadian rhythm are often linked to abnormalities in the circadian locomotor output cycles kaput gene. These abnormalities can affect the proper functioning of the suprachiasmatic nuclei (SCN), which serve as the body’s internal pacemakers. Consequently, these disturbances in the SCN can contribute to poor sleep quality among individuals with depression (54).

Moreover, heightened sleep reactivity due to stress is a common occurrence in various mental health disorders. This heightened reactivity refers to an increased sensitivity to environmental or internal stimuli that can disrupt sleep. Individuals with mental health conditions, including depression, often experience difficulties falling asleep or staying asleep due to heightened reactivity to stressors, further impacting their sleep quality (54).

### Limitation of the study

The Pittsburgh Sleep Quality Index (PSQI) is primarily used to screen and identify poor sleep quality rather than diagnose specific sleep disorders. It assesses various sleep aspects over a defined period, offering insights into sleep habits. However, it does not diagnose specific sleep disorders or underlying causes. Its single-time assessment provides a snapshot of sleep quality, offering a general overview rather than tracking changes over time.

## Conclusion

Almost 1 out of 2 CKD patients assessed for poor sleep quality tested positive. It was noted that poor sleep quality was more frequent among CKD patients with common mental disorders, anemia, decreased eGFR levels, individuals aged over 50 years, and those with a longer duration of the disease. Consequently, it’s advised to regularly screen these CKD patients for sleep quality.

## Data availability statement

The original contributions presented in the study are included in the article/supplementary material, further inquiries can be directed to the corresponding author.

## Ethics statement

The studies involving humans were approved by University of Gondar Institutional Review Board. The studies were conducted in accordance with the local legislation and institutional requirements. The participants provided their written informed consent to participate in this study.

## Author contributions

YG: Conceptualization, Data curation, Formal analysis, Investigation, Methodology, Software, Writing – original draft, Writing – review & editing. LL: Writing – original draft, Writing – review & editing. WS: Writing – original draft, Writing – review & editing. WA: Writing – original draft, Writing – review & editing. GC: Writing – original draft, Writing – review & editing. AS: Writing – original draft, Writing – review & editing. AB: Writing – original draft, Writing – review & editing. DE: Writing – original draft, Writing – review & editing. MMi: Writing – original draft, Writing – review & editing. AM: Writing – original draft, Writing – review & editing. MMe: Methodology, Writing – original draft, Writing – review & editing.
